# Mammosphere Formation in Breast Carcinoma Cell Lines Depends upon Expression of E-cadherin

**DOI:** 10.1371/journal.pone.0077281

**Published:** 2013-10-04

**Authors:** Juan Manuel Iglesias, Izaskun Beloqui, Francisco Garcia-Garcia, Olatz Leis, Alejandro Vazquez-Martin, Arrate Eguiara, Silvia Cufi, Andres Pavon, Javier A. Menendez, Joaquin Dopazo, Angel G. Martin

**Affiliations:** 1 Regulation of Cell Growth Laboratory, Fundacion Inbiomed, San Sebastián, Gipuzkoa, Spain; 2 Computational Genomics Institute, Centro de Investigación Principe Felipe (CIPF), Valencia, Spain; 3 Functional Genomics Node, INB, CIPF, Valencia, Spain; 4 Translational Research Laboratory, Catalan Institute of Oncology (ICO), Girona, Spain; 5 Girona Biomedical Research Institute (IDIBGi), Girona, Spain; 6 Centro de Investigación Biomédica en Red de Enfermedades Raras (CIBERER), Valencia, Spain; Institute of Medical Science, University of Tokyo, Japan

## Abstract

Tumors are heterogeneous at the cellular level where the ability to maintain tumor growth resides in discrete cell populations. Floating sphere-forming assays are broadly used to test stem cell activity in tissues, tumors and cell lines. Spheroids are originated from a small population of cells with stem cell features able to grow in suspension culture and behaving as tumorigenic in mice. We tested the ability of eleven common breast cancer cell lines representing the major breast cancer subtypes to grow as mammospheres, measuring the ability to maintain cell viability upon serial non-adherent passage. Only MCF7, T47D, BT474, MDA-MB-436 and JIMT1 were successfully propagated as long-term mammosphere cultures, measured as the increase in the number of viable cells upon serial non-adherent passages. Other cell lines tested (SKBR3, MDA-MB-231, MDA-MB-468 and MDA-MB-435) formed cell clumps that can be disaggregated mechanically, but cell viability drops dramatically on their second passage. HCC1937 and HCC1569 cells formed typical mammospheres, although they could not be propagated as long-term mammosphere cultures. All the sphere forming lines but MDA-MB-436 express E-cadherin on their surface. Knock down of E-cadherin expression in MCF-7 cells abrogated its ability to grow as mammospheres, while re-expression of E-cadherin in SKBR3 cells allow them to form mammospheres. Therefore, the mammosphere assay is suitable to reveal stem like features in breast cancer cell lines that express E-cadherin.

## Introduction

The cancer stem cell model of tumor growth gives us a framework to explain the intra-tumor heterogeneity observed in tumors and is supported by the fact that only a specific subset of cancer cells within the original tumor are able to propagate tumor growth, when transplanted into immunosuppressed mice, resembling the heterogeneity displayed by the original tumor [1]. In many ways cancer stem cells (CSCs) are similar to normal stem cells: both types of cells share the self-renewal ability and they are able to generate differentiated descendants. CSCs are likely responsible for tumor growth, metastatic expansion of the tumor and relapse after surgery or chemotherapy. Despite their role as central players in cancer biology, our knowledge about their biology and origin is still very limited. CSCs may arise from normal tissue stem cells harboring transforming mutations or from more differentiated cells that during tumor progression acquire stem cell traits [2]. Breast cancer cells with a CD44^+^/CD24^low/-^ surface phenotype were found to have tumor-initiating properties with stem-cell like features and invasive ability [3], however it is unclear whether their presence in a tumor has clinical implications [4]. Furthermore, CD44^+^/CD24^low/-^ cells are more frequent in basal breast tumors (and particularly high in BRCA1 mutated tumors) suggesting that the cancer stem cells are not restricted to those markers [5]. Although there is no definitive consensus on the phenotype and frequency of CSCs in the majority of human solid tumor types, enough experimental evidence supports that many tumors of both epithelial and non-epithelial origin have functionally defined CSCs and that it affects tumor biology [6] [2].

The mammosphere assay was developed as a method to propagate mammary epithelial stem cells (MaSC) in vitro by Dontu et al. [7] as a modification of the neurosphere assay developed by Reynolds et al. [8] This assay has been used as a surrogate reporter of stem cell activity in the mammary gland [9] and cancer stem cell activity [10]. The assay is based on the premise that only undifferentiated cells derived from the mammary epithelium will survive in suspension culture with all the other cell types dying by anoikis. The ability to form several generations of mammospheres in serial non-adherent passage is related to the self-renewal ability of the stem cells giving rise to these structures. Probably one of the biggest limitations of this culture system, as a way to maintain and propagate human MaSCs, is that after a few (not more than 5) passages in suspension the culture extinguishes [11] [12], so the self-renewal potential of human MaSCs seems to be exhausted after these number of passages, when maintained in these culture conditions. Whether there is a technical limitation imposed by incomplete understanding of culture requirements or a perpetual self-renewal barrier, limiting the expansion of normal stem cells in the tissues, is a matter of active discussion. This in vitro culture system also proved to be useful for the selection and propagation of tumorigenic breast cancer cells from primary tumors [13] and metastasis [14] and even as a tool to screen for new drugs targeting CSCs [15]. Therefore, we need to be careful about the interpretation of this assay when used to measure cancer stem cell activity within a tumor.

Epithelial to Mesenchymal Transition (EMT) is a critical program that mediates tumor invasion and metastasis. This program is mediated by the activity of transcription factors such as SNAIL 1/2, ZEB 1/2 or TWIST 1/2, which results in loss of E-cadherin (E-Cad) expression, loss of cell polarity, acquisition of loose mesenchymal cell morphology and invasion capabilities, critical for the metastatic spread of epithelial tumors. In recent years it has been shown that EMT is also involved in cellular plasticity, a process by which non stem cells acquire stem cell traits. Mani et al. [16] found that induction of EMT in immortalized human mammary epithelial cells increased the expression of stem cell markers, and their ability to form mammospheres. On the other hand, mesenchymal and stem cell traits are selected in breast cancer cells lines when grown in suspension culture [17] [18]. Expression of EMT markers like vimentin increases with the passage number in suspension culture. Besides, previous work from our group has shown that the expression of SOX2, a key stemness transcription factor, changes when breast cancer cell lines are maintained in suspension culture, the higher the passage number, the higher the number of SOX2 positive cells in the spheres [19].

In an effort to apply the mammosphere assay to measure self-renewal to a wide variety of breast cancer cell lines, we tested the ability of eleven human breast cancer cell lines representing the major breast tumor subtypes, to grow as mammospheres. We found that several cell lines (MCF7, T47D, BT474, MDA-MB-436 and JIMT-1) had the ability to grow as mammospheres through serial non-adherent passage, while other cell lines did not. Based on this feature we can distinguish three groups of cell lines: the long term sphere forming cell lines (-LTSF- MCF7, T47D, BT474, MDA-MB-436 and JIMT-1), the short term sphere forming cell lines (-STSF- HCC1937 and HCC1569) and the non sphere forming cell lines (-NSF- MDA-MB-231, MDA-MB-435, MDA-MB-468 and SKBR3). We performed a meta-analysis of gene expression data to identify putative regulators of this phenotype. We found that genes related to cell motion and migration are overrepresented among the genes differentially expressed in the sphere forming cell lines. Further analysis at the protein level showed a strong correlation between E-cadherin expression and sphere formation ability. Silencing of E-cadherin expression in MCF7 cells, a LTSF cell line, prevented the formation of mammospheres, while overexpression of E-cadherin in non-spherogenic cells (SKBR3) resulted in sphere formation. Consequently, the ability to grow as spheroids depends on E-cadherin expression. Therefore, the mammosphere assay may be a useful assay to study stem cell like behavior in some breast cancer cell lines.

## Materials and Methods

### Cell lines and culture conditions

MCF-7, T47D, MDA-MB-231, MDA-MB-435, MDA-MB-468 breast carcinoma cell lines was obtained directly from the ATCC (Manassas, VA, USA), JIMT-1 cells were obtained from the German Collection of Microorganisms, MDA-MB-436 and HCC-1937 were obtained from T. Stein (U. of Glasgow, UK, previously obtained from ATCC, Manassas, VA, USA) and SKBR3, BT474 and HCC-1569 were obtained form A. Pandiella (CIC-Salamanca, Spain, previously obtained from ATCC, Manassas, VA, USA) and grown in DMEM (Gibco, Carlsbad, CA) supplemented with 10% fetal bovine serum (FBS, Sigma, St. Louis, MO) at 37°C in a 5% CO_2_ incubator. Characterization of cell lines according to their surface expression phenotype can be found in Figure S1. For mammosphere formation, single cell suspensions were plated in 6-well tissue culture plates covered with poly-2-hydroxyethyl-methacrylate (Sigma, St. Louis, MO) to prevent cell attachment, at a density of 1,000 cells/ml in serum-free DMEM supplemented with 1% L-glutamine, 1% penicillin/streptomycin, 30% F12 (Sigma), 2% B27 (Invitrogen, Carlsbad, CA), 20 ng/ml EGF (Sigma, St. Louis, MO) and 20 ng/ml FGFb (Invitrogen, Carlsbad, CA). The medium was made semi-solid by the addition of 0.5% Methylcellulose (R&D Systems, Minneapolis, MN) to prevent cell aggregation. After 7 days in culture, mammospheres were collected by gentle centrifugation (200 x g) and dissociated enzymatically (5 min in 1:1 trypsin/DMEM solution at 37°C) and mechanically by passing through a 25G needle (6 strokes). Single cells were re-plated at a density of 1,000 cells/ml for subsequent passages.

### Immunofluorescence

Mammospheres were collected by centrifugation in a Cytospin (Thermo, Waltham, MA, USA) onto glass slides prior to fixation. For monolayer cultures, cells were grown on LAB-TEK II chamber slides (Nalgene Nunc International, Penfield, NY). Cells were fixed in 4% paraformaldehyde for 15-30 min at room temperature, washed with PBS and permeabilized/blocked for 1h at room temperature with PBS, 0.3% Triton X-100, 2.5% horse serum. Slides were then incubated with anti-E-cadherin primary antibody (diluted 1:100) (see File S1) overnight, at 4°C washed in PBS. Appropriate fluorophore labeled secondary antibody was added at a dilution of 1/250 and incubated for 1 h at room temperature, and after washing in PBS, Hoescht 33342 dye was added to reveal nuclear DNA. Immunofluorescence was visualized in a Zeiss LSM510 confocal laser-scanning microscope.

### Microarray data analysis

#### Data was standardized using background correction and quantile normalization [20]

Differential gene expression was carried out using the limma [21] package from Bioconductor (http://www.bioconductor.org/). We performed a statistical test for each probe. This microarray includes 33078 probes and we were in a multiple comparison scenario, so we had to apply a multiple testing adjustment of p-values to avoid detecting false positives. This process was done according to Benjamini and Hochberg [22] methodology.

Gene set analysis was carried out for the Gene Ontology terms using FatiScan [23] in Babelomics [24] (http://babelomics.bioinfo.cipf.es/). This is a web-based program for the functional interpretation of large-scale experiments. The test implemented aims to directly test the behaviour of blocks of functionally related genes, instead of focusing on single genes. This tool detects significantly up- or down-regulated blocks of functionally related genes in lists of genes ordered by differential expression. FatiScan returns adjusted p-values based on False Discovery Rate (FDR) method [25], [22].

Significant GO terms were represented by directed acyclic graphs from Blast2GO [26].

GO annotation for the genes in the microarray where taken from Ensembl 56 release (http://www.ensembl.org).

### Gene Silencing and overexpression

Pre-packaged lentiviral particles that either encoded a non-targeting shRNA (negative shRNA, sc-108080) or sequences specifically targeting the human E-cadherin gene [E-cadherin shRNA(h) lentiviral particles, sc-35242-V] were purchased from a commercial provider (Santa Cruz Biotechnology). For viral infection of MCF-7 cells, the regular medium was replaced with culture medium containing 5 µg/mL polybrene (Santa Cruz Biotechnology, sc-124220). MCF-7 cells were then exposed to lentiviruses for 48 h. Because the lentiviral shRNA particles also encode a puromycin resistance gene for transduction selection, the cells were then washed and grown in culture medium containing 10 µg/mL puromycin dihydrochloride (Sigma, P9620) for an additional 72 h. The transduced MCF-7 cells were allowed to recover and proliferate for at least 1 week before any experimental procedures and were then analyzed. To monitor the lentiviral transduction efficiency and transgene expression for the duration of the experiment, we incubated additional subsets of MCF-7 cells with lentiviral particles encoding a green fluorescence protein (GFP) reporter (sc-108084). Transduction efficiency (> 90%) was obtained as the ratio of the number of GFP-positive cells to the total number of cells from five random visual fields from three independent culture experiments.

Stable cell lines expressing E-cadherin were generated by infection with the lentiviral vector pWZL Blast mouse E-cadherin (Addgene plasmid 18804) [27], infected cells were selected using 0.5µg/ml of blasticidine.

### Flow cytometry

The cells were harvested by trypsinization into single-cell suspensions, washed with staining buffer (PBS, 3% FBS and 0.03% sodium azide), counted and resuspended in staining buffer plus 10% FBS at a cell density of 10^6^ cells/100 µl for 10 min at 4°C. Cell suspensions were spun down and the different combinations of fluorochrome-conjugated monoclonal antibodies (see File S1 for antibody list) or their respective isotope controls (recommended by the manufacturer) were added to the cell suspension at the concentrations recommended by the manufacturer in staining buffer and incubated in the dark at 4°C for 45 min. Labeled cells were washed 3 times with staining buffer and analyzed using a BD FACSCanto or FACSCalibur (BD Biosciences). FlowJo (Tree Star, Inc) was used to analyze the data.

## Results

### Proliferation kinetics of human breast cancer cell lines growing in suspension culture

**Figure 1:**
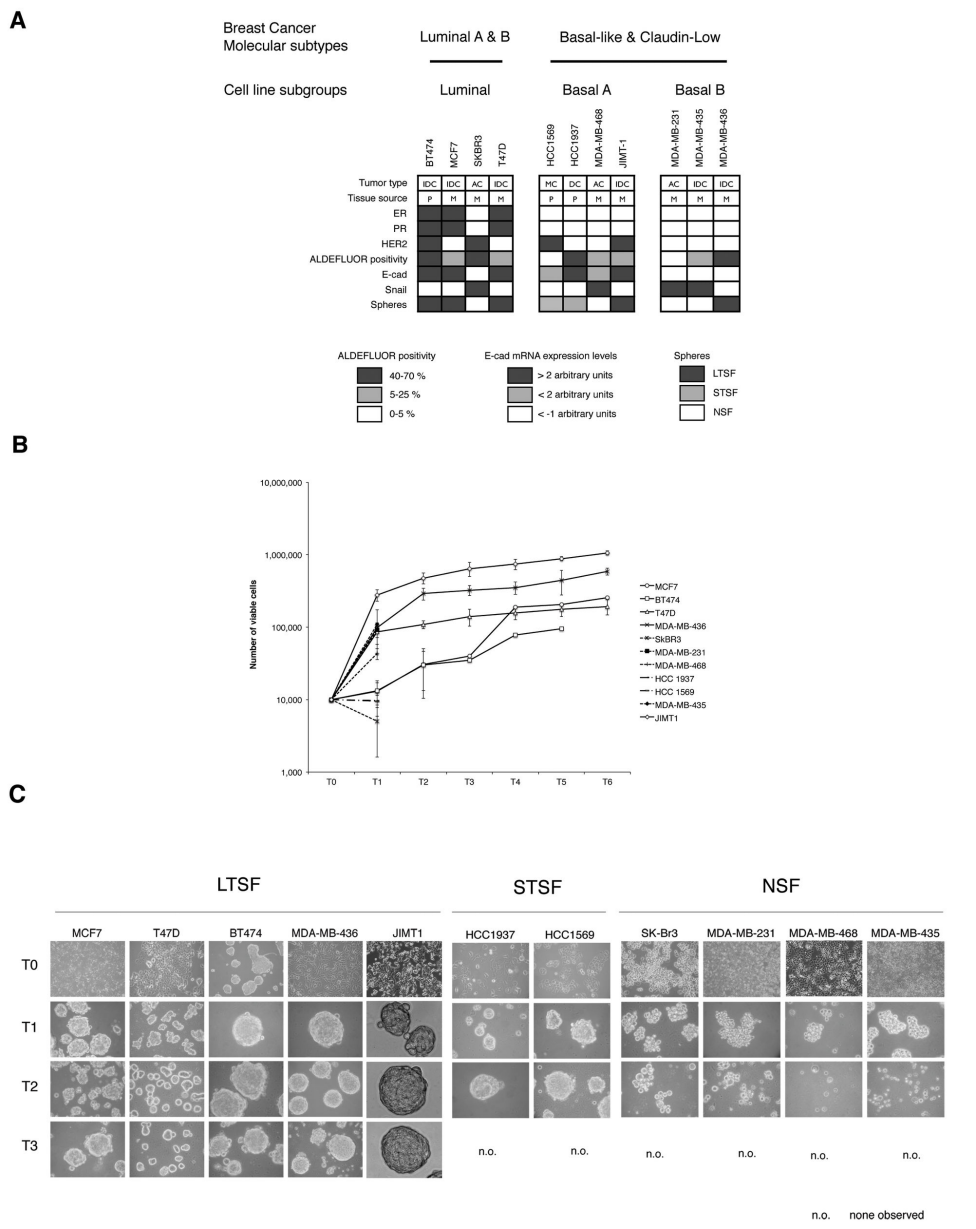
Breast cancer cell lines characteristics in suspension culture. A) Breast cancer cell lines included in the study showing their origin and main molecular characteristics (IDC, invasive ductal carcinoma; AC, adenocarcinoma; DC, ductal carcinoma; P, primary tumor; M, metastasis; black box, positive; white box, boxes color code stated in the figure). B) Proliferation of breast cancer cell lines in suspension culture. Cell proliferation was measured as the increase in the number of viable cells upon serial non-adherent passages. C) Morphology of the human breast cancer cell lines included in the study. The morphology of the cell lines growing in adhesion is shown in the top row, indicated as T0. Subsequent passages in suspension culture are termed T*n*, where n represents the passage number (Magnification 120X).

In order to test the growth properties of breast carcinoma cells maintained in suspension culture, we used the mammosphere assay as described originally by Dontu et al. [7] Eleven human breast cancer cell lines were selected to represent the major molecular subtypes of breast cancer (Figure 1A, for cell line characterization see Figure s1) and were cultured in suspension on non-adherent plates to test their ability to grow as mammospheres. To study their proliferation kinetics all cell lines were seeded in DMEM:F12 (2:1) medium without serum, supplemented with B27, EGF (20 ng/ml), bFGF (20 ng/ml). In order to prevent cell aggregation, methylcellulose was included in the medium at a final concentration of 0.5%. All the cell lines were seeded at a cellular density of 10^3^ cells/ml, at this cellular density the sphere assay gave rise to clonal spheres as described in other systems [28]. In our hands, using this seeding conditions, we obtain roughly the same frequency of sphere initiating cells (see ref [29]) as compared to a quantitative limited dilution assay (Figure S2). Serial mechanical and enzymatic passage was performed every 7 days, as described in the methods section. Number of viable cells was recorded and plotted against passage number (Figure 1B). In these growth conditions only the cell lines MCF7, T47D, BT474, MDA-MB-436 and JIMT-1 (Long Term Sphere Forming –LTSF- cell lines from now on) could be propagated beyond passage 6 (14, 7, 11, 10 and 12 passages, respectively, for MCF-7, T47D, BT474, MDA-MB-436 and JIMT-1). The LTSF cell lines formed smooth and round spheres when grown on suspension culture (Figure 1C) that needed to be enzymatically and mechanically disaggregated in order to obtain a single cell suspension before subculturing.

The viability of the cell lines SKBR3, MDA-MB-231, MDA-MB-468 and MDA-MB-435 dropped dramatically on passage 2 and they could not be propagated any further in suspension culture. Those cell lines were termed Non-Sphere Forming –NSF- cell lines as they formed loosely adhered clumps of cells (Figure 1B) that can be easily dispersed by pipetting. These were not considered proper mammospheres.

The cell lines HCC1937 and HCC1569 did form spheroids that needed to be disaggregated through enzymatical and mechanical treatment and morphologically they resemble the mammospheres generated by the LTSF cell lines (Figure 1C). However, no viable cell was recovered after passage 2, therefore they were termed Short Term Sphere Forming –STSF- cell lines. Therefore, 3 groups of cell lines can be distinguished depending on their ability to form spheroids and their capability to be grown as long term suspension cultures: LTSF, STSF and the NSF cell lines.

### Characterization of LTSF, STSF and NSF cell lines

The NSF and STSF cell lines fall mainly into the basal subtype with the only exception of SKBR3 cells that are classified as luminal and show HER2 amplification (Neve et al. [30]). Both STSF cell lines are classified as basal A although they harbor distinct features, HCC1937 are BRCA1 mutated (5382insC) [31] and HCC1569 show HER2 amplification. The LTSF group is a heterogeneous group with 3 out of 5 cell lines being luminal. MCF7, BT474 and T47D are prototypical luminal cell lines with BT747 cells harboring an amplification of the HER2 gene. On the other hand MDA-MB-436 and JIMT1 are classified as basal and both harbor distinct features, MDA-MB-436 are BRCA1 mutated (5396 +1G>A) [31] and the JIMT1 show HER2 amplification. All the breast carcinoma cell lines included in the study are tumorigenic when injected in immunodeficient mice.

As the sphere-forming assay relates to stem cell features, we tested the expression of several stem cell markers described for breast cancer stem cells. The expression of the stem cell markers CD44 and CD24 was tested by FACS in all cell lines included in the study (Figure S3) alongside other surface markers. We did not find a direct correlation between the ability to form spheroids and the CD44^high^/CD24^-/low^ population, breast cancer stem cell phenotype, in the parental culture. Besides, high Aldehyde Dehydrogenase 1 (ALDH1) activity measured through the Aldefluor**®** assay, another described marker for stem cell population, did not correlate with the ability of the cell lines to grow as mammospheres (Figure 1A and Figure S4). The expression of other surface markers sometimes associated to stem cell activity (CD49f, EpCAM, CD29, CD10 and Muc1) did not correlate with sphere formation ability (Figure S1). Altogether, expression of stem cell associated markers in the parental cell line population did not correlate with the ability to form long-term sphere cultures.

### Meta-analysis of genes differentially expressed between the LTSF, STSF and NSF groups

Gene expression analysis was performed looking for genes differentially expressed between the LTSF, STSF and NSF groups using the public dataset GSE15361 from Neve et al. [30]. This dataset includes the expression profile from 53 different breast cancer cell lines. JIMT-1 gene expression profile was not included in our analysis as data from this cell line are not available. Due to the conflicting results reported in the literature [32], MDA-MB-435 expression profile was not included in the analysis neither. The expression profile for each of the cell lines included in our study were downloaded, normalized and re-analyzed using Bioconductor [33] and Babelomics [24]. Using this strategy 44 genes were found differentially expressed between the 3 groups with a p value <0.001 (see File S2 for a detailed annotation of differentially expressed transcripts).

**Figure 2:**
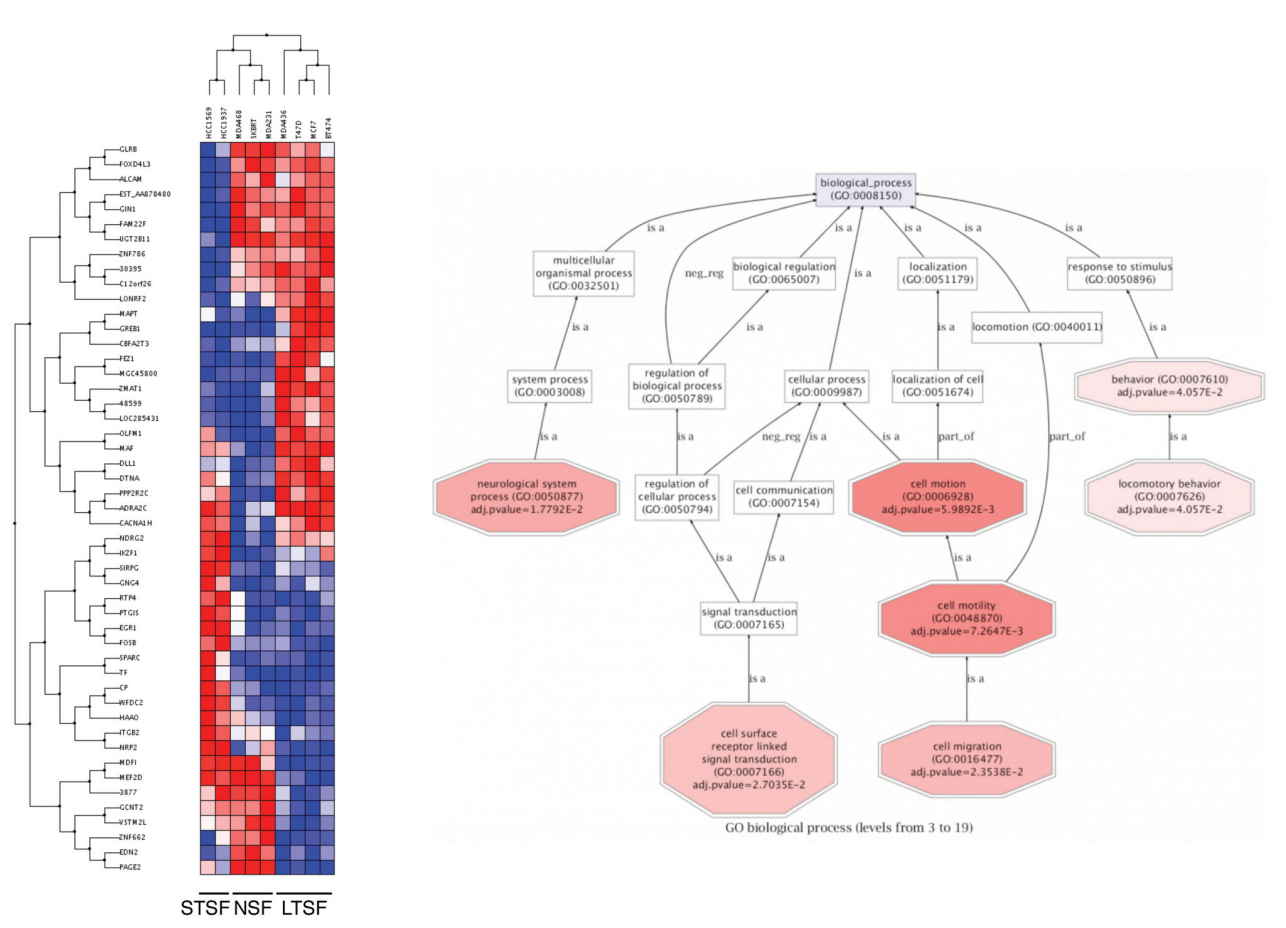
Gene expression profiling of the breast cancer cell lines included in the study. Left panel: clustering of the genes differentially expressed (p-value < 0.001) between the 3 groups of cell lines identified based on their proliferation and morphological characteristics in suspension culture. Blue squares represent transcript levels below the median; white squares represent transcript levels equal to the median; red squares represent transcript levels greater than the median. Right panel: functional annotation of biological processes overrepresented among the genes differentially expressed between the three groups of cell lines.

**Figure 3:**
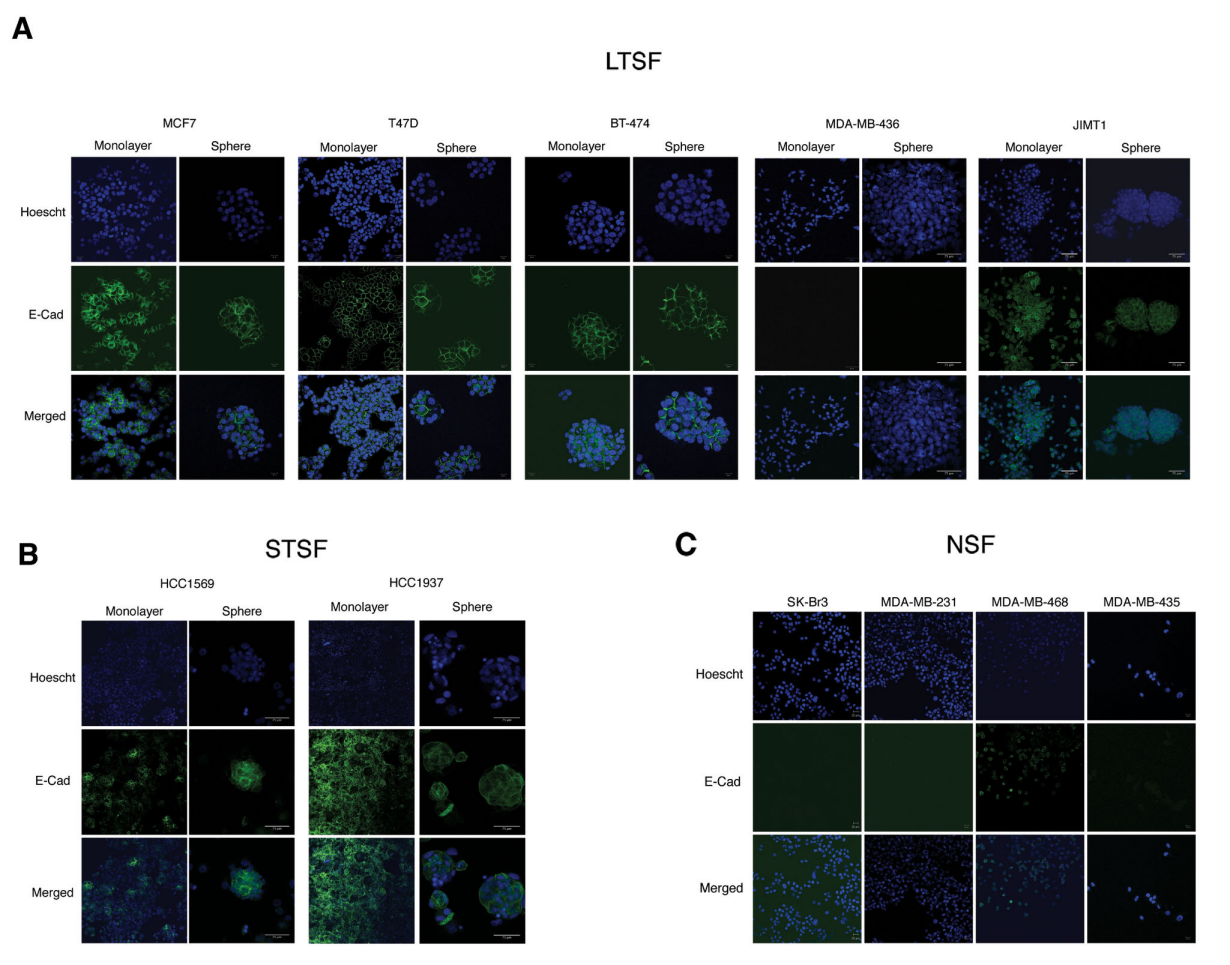
E-cadherin expression in LTSF (A), STSF (B) and NSF (C) breast cancer cell lines growing in adhesion and suspension culture assessed by immunofluorescence.

Clustering of these genes allows the identification of three groups of genes differentially regulated in LTSF, STSF or both compared to NSF cell lines. These genes may be related to the ability of these cell lines to grow as spheroids when grown in suspension culture. GO analysis (Figure 2) showed enrichment in genes related with cell motion and cell migration, one of the hallmarks of EMT. During EMT cells acquire mesenchymal traits and become more motile, a property that is key for tumor cells to become metastatic. Therefore we decided to look at the changes in expression of different EMT drivers and markers within the data generated in our microarray analysis. At the transcriptional level we found that E-cadherin mRNA expression correlates with sphere formation and this correlation was further confirmed at the protein level by IF (Figure 3). All LTSF cell lines but MDA-MB-436 show uniform E-cadherin expression and it is localized to the cell-cell junctions, as expected when cells are grown in adhesion or suspension cultures. On the STSF group HCC1569 cells show patched expression in adhesion culture, though all the cells on the spheroids express E-cadherin at the cell-cell junctions. Among the NSF group only MDA-MB-468 cells express E-cadherin in the adhesion cultures with individual cells stained, but we failed to identify epithelial islands with E-cadherin localized to the cell-cell junctions as found in HCC1569.

Therefore all the cell lines growing as mammospheres (LTSF or STSF) express E-cadherin in adhesion and suspension cultures with the only exception of MDA-MB-436, this cell line does not express E-cadherin but grow as mammospheres and long-term cultures can be maintained.

### E-cadherin expression determines the ability to form spheres

**Figure 4:**
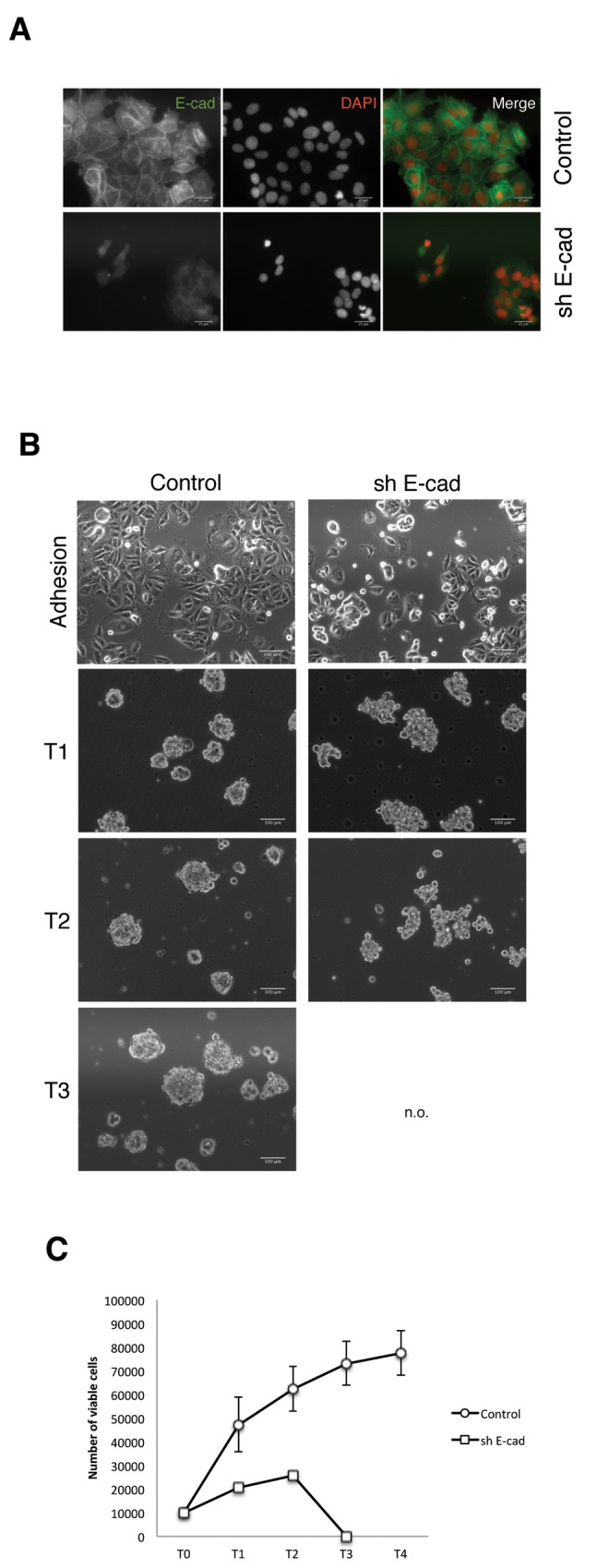
E-cadherin knock-down in MCF7 cells inhibits mammosphere formation. A) Immunofluorescence staining of MCF7 shE-cad and control cells to show the knockdown of E-cadherin expression. B) Morphology of MCF7 shE-cad and control cells growing in adhesion and suspension culture. C) Proliferation of MCF7 shE-cad and control cells in suspension culture. Proliferation was measured as the increase in the number of viable cells upon serial non-adherent passages. The plot was made using data from 3 independent experiments. N.O.=Non observed.

**Figure 5:**
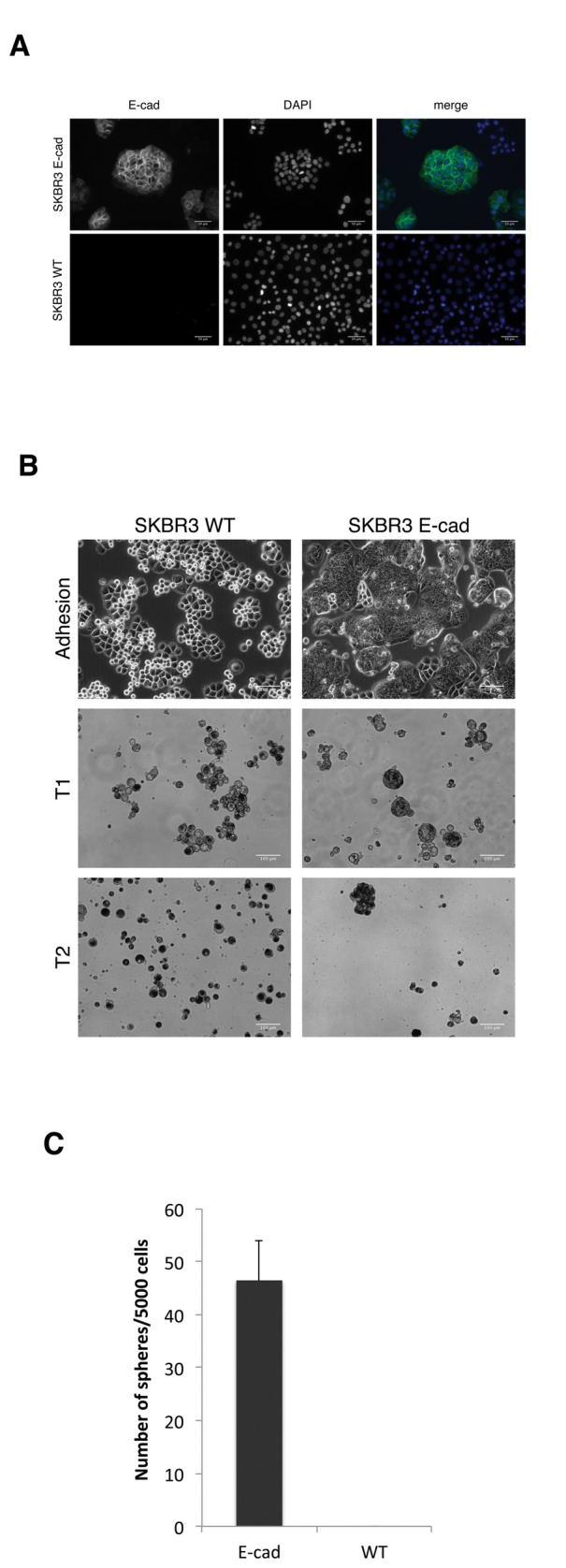
Expression of E-cadherin in SKBR3 cells induces sphere formation. A) Immunofluorescence staining of SKBR3 E-cadherin and WT cells showing the expression of E-cadherin. B) Morphology of SKBR3 E-cadherin and WT cells growing in adhesion and suspension culture. C) Quantitation of the number of spheres made by SKBR3 E-cadherin and WT cells in T0. The plot was made using data from 3 independent experiments.

To test whether E-cadherin expression is necessary for the cells to grow as mammospheres two different approaches were taken: on one hand, we knocked down E-cadherin expression in MCF-7 (a LTSF cell line) through shRNA mediated gene silencing. The knock down of E-cadherin in MCF-7 cells blocked their ability to grow as long-term suspension cultures and their ability to form spheroids, as show in Figure 4. MCF7 cells in which E-cadherin expression was knocked down grew as loosely adhere clumps in suspension culture, thus behaving as a NSF cell line. On the other hand, SKBR3 cells, which are classified by their intrinsic expression profile as a luminal breast cancer cell line [30], harbor a homozygous deletion of E-cadherin [34]. Thus, we re-expressed E-cadherin in SKBR3 cells, what then allowed them to grow as mammospheres in suspension culture. The SKBR3 cells expressing E-cadherin did form mammospheres and they behaved as a STSF cell line, since they only survived for one passage in suspension culture (Figure 5).

These functional assays are in accordance with the observation that E-cadherin expression correlates with sphere formation, but not with the self-renewal potential of the suspension cultures.

## Discussion

The mammosphere assay was originally developed by Dontu et al. [7] as a way to select for and propagate MaSCs. Soon after the publication of this assay researchers started to use it as a reporter of stem cell and cancer stem cell activity from tissue samples, tumors and continuous cell lines [13] [14] [35]. Breast carcinoma cell lines represent clonal outgrowths that have survived the selection pressures of in vitro culture and, as such, do not fully represent clinical cancers growing in situ. They are easily accessible cellular models to study the molecular mechanisms of tumorigenesis and it has been shown that most of these cell lines contain a small subpopulation of cells displaying functional stem cell characteristics with tumorigenic capacity [35]. Theoretically, continuous tumor cell lines may contain a population of stem-like cells as they are capable of initiating tumors in immune-compromised animal models and survive passage after passage, otherwise cell cultures would eventually exhaust. In fact, all the cell lines included in the study are tumorigenic when injected in immunodeficient mice, therefore all of them should contain cells with stem-like characteristics and tumor initiating capacity. Currently, the xenograft tumor initiation assay is the gold standard assay to measure stemness.

Besides, FACS has been used to enrich for CSCs using different combinations of surface markers (CD24, CD44, CD49f, CD29), or fluorescent substrates such as Aldeflour**®** (that measures ALDH activity, augmented in normal stem cells and CSCs) or DNA dyes (such as Hoescht for measuring side population), although there is not a good marker reported so far to identify breast CSCs described, with multiple marker configurations that identify cell populations that do not overlap. These subpopulations of cells with stem cell characteristics show higher frequency of mammosphere initiating cells in clonogenic assays, as compared to the parental cell line. However, recent reports show that there does not necessarily exist a clear correlation between the expression of stem cell markers by the parental cell line and its sphere formation ability, since cell lines with no detectable ALDH activity [36] or lacking a CD44^+^/CD24^-/low^ population (see Figure S3) can be propagated in suspension culture for several passages. Sarrio et al. [37] recently demonstrate that in continuous cell lines this marker profile on its own does not identify a stem cell population with the ability to restore the heterogeneity of the parental cell line. Moreover, they also report that in MCF12A mammary epithelial immortalized cells, the CD44^+^/CD24^-/low^ profile, found to enrich for cancer stem cells in primary breast tumors [3], failed to enrich for mammosphere initiating cells [38]. Breast carcinoma cell lines show a homogenous surface marker expression pattern, as opposed to fresh tumor cells, not evidencing the presence of particular subpopulations at surface marker level. When used to evaluate tumor initiation of common breast carcinoma cell lines, the percentage of CD44^+^/CD24^-^ did not correlate to tumorigenicity [35], it does, however, correlate strongly with rare basal and mesenchymal phenotype [39]. Therefore, the CD44^+^/CD24^-/low^ profile clearly associates with a mesenchymal-like phenotype but does not identify the cell lines with sphere forming ability. This correlation is even stronger if only CD24 expression is taken into consideration [40], for example, MCF7, BT474 or T47D, all LTSF cell lines, show 100% expression of CD24 (and CD44 med/low). These data suggests that there is not a clear correlation between the ability to grow as spheroids and the expression of reported stem cell markers. This may be due to the lack of suitable markers for the study of breast cancer stem cells or to the intrinsic plasticity of the tumor epithelial cells. This likely reflects the lack of understanding about the markers expressed along the human mammary epithelial hierarchy, unlike in the leukaemia field where cancer stem markers where selected based on the markers expressed by the well-studied normal hematopoietic lineage. Another possibility contributing to the lack of reliable markers to identify breast cancer stem cells is the intrinsic cellular plasticity of the tumor cells, as cells initially lacking stem cell activity may gain this capability without a reflection on the surface marker profile.

We base our work on the in vitro mammosphere assay as a functional assay reported to enrich for cells with enhanced tumor initiation ability in breast carcinoma cell lines [13] [14] and thus prevent marker bias. Previous work from our group demonstrated that the expression of the stemness factor Sox2 is linked to the ability of LTSF cell lines to form spheroids and the tumorigenicity of these cell lines [19], thus validating the use of this assay as a suitable tool for the selection of cells with cancer stem cell traits.

In our hands, continuous breast carcinoma cell lines such as MDA-MB-231, failed to be propagated as mammosphere cultures, when other authors state they form spheroids [41] [42]. These differences may be due to the use of different culture conditions, such as different cellular density at the moment of seeding, different medium composition and the lack of standardized morphological criteria. A recent review on the neurosphere assay shows that cell density affects the ability of the cells to grow as spheroids, making it difficult to distinguish cell aggregation from spheroid formation [43]. In our experience, the addition of methylcellulose to the culture media and the use of low seeding density (no higher than 10^3^ cells/ml) ensures that spheroids have a clonal origin [28]. The amount of spheres rendered using this seeding conditions is consistent with quantitative limiting dilution assays that measure the frequency of sphere forming cells in the original cell population (see Figure S2). An important point when using this in vitro suspension culture system to study stemness-associated properties is to check the viability upon serial suspension culture as a way to monitor the exhaustion of their self-renewal potential. From the morphological point of view, our criteria to decide whether a floating structure is a cell aggregate or a spheroid is that the cell aggregates are easily dissociated by mild mechanical pipetting up and down, while spheroids, in order to be efficiently dissociated, need to be enzymatically and mechanically disaggregated. This behavior likely reflects the lack of proper cell-cell interactions in NSF, as absence of expression of typical cell adhesion molecules such as E-cadherin.

While the exhaustion of the self-renewal potential of normal mammary epithelial stem cells takes place after 4-5 passages in suspension culture, there is much more variability when working with cancer stem cells, either derived from tumor samples or continuous cell lines. In general, fresh tumor cells cannot be grown in these culture conditions for longer than 4-5 passages, although Ponti et al. [13] reported the isolation of cell lines from pleural effusions that can be maintained in suspension culture for over 40 passages. We found that LTSF cell lines can be maintained in suspension culture for at least 6 passages but no longer than 14, on the other hand STSF and NSF cells lines stop proliferating after just 1-2 passages. Therefore, we propose cell viability and enzymatic disaggregation upon serial passages as criteria to distinguish true spheroid formation.

Our results support an association between epithelial phenotype, as opposed to mesenchymal-like (EMT-associated) motile phenotype, and spheroid formation. Our meta-analysis of gene expression data comparing LTSF and STSF versus NSF cell lines revealed a significant difference in the expression of genes associated with cell motility and migration, particularly at the level of the cell surface. We have uncovered a functional relation between E-cadherin expression, associated with typical epithelial morphology, and spheroid formation. E-cadherin is one of the earliest cell surface molecules that is down-regulated during EMT, allowing the cells to escape physical constraints imposed at the epithelium, acquire a mesenchymal-like phenotype and invade other tissues, the first step towards tumor dissemination. NSF cell lines (mesenchymal in phenotype) do not express E-cadherin on the cell surface, while LTSF and STSF do express E-cadherin, readily detectable at the cell-cell contact interface (Figure 3). Interestingly, the intensity of cohesion among the cells themselves has been reported to determine motility or adhesion of individual cells, as the physical and molecular basis of adhesion based morphogenetic phenomena, with expression of classical “type I” cadherins as critical determinants of this phenomenon [44] [45]. Therefore, it is the expression and type of homotypic cadherin molecules, besides the ability of a particular cell to manifest stem-like activity, which will allow cell association in spheroid formation. When E-cadherin gene was silenced in a typical LTSF cell line (MCF7), cells rapidly acquired a loose (mesenchymal-like) morphology and they no longer showed the ability to grow as spheroids. Moreover, the cell line SKBR3, that presents mesenchymal morphology despite showing a luminal epithelial gene signature, and classified in our study as NSF cell line, presents a homozygous deletion in the region that contains the E-cadherin gene. When expression of E-cadherin was reconstituted, these cells now made spheroids successfully. EMT has been proposed to induce stem cell features in breast cancer cells [16], though in our hands typical mesenchymal breast carcinoma cell lines are not capable of forming spheroids in vitro. The MDA-MB-436 mesenchymal breast carcinoma cell line is an outlier in this classification, as it is capable of forming *bona fide* sphere structures (using the viability and disaggregation criteria proposed above), but do not express E-cadherin (Figures 1 and 3). A deeper molecular profiling must be carried out in order to understand cell surface expression profile in this cell line.

In summary, we find that the ability to form spheroids correlates with the acquisition of epithelial phenotype and expression of E-cadherin. In accordance with our results, Celia-Terrasa et al. [46] have recently characterized two cellular models, derived from prostate and bladder cancer cell lines, where forced induction of EMT in subpopulations displaying stable epithelial features, resulted in the suppression of cancer stem cell properties, including anchorage-independent growth and metastatic potential. Overexpression of the EMT-promoting factors SNAIL1, TWIST1 and TWIST2 reduced the ability of the prostate cancer cell line PC3 to form spheroids. Conversely, knockdown of E-cadherin led, in addition to a loss of their epithelial features, to inhibition of cancer stem cell properties such as spheroid formation and their capacity to colonize distant organs in vivo. On the other hand, when a high E-cadherin expression subpopulation of the prostate cancer cell line PC-3 was sorted out, these cells showed increased spheroid formation compared with the parental population. Knockdown in these cells of SNAI1, ZEB1 and TWIST2 caused a loss of their EMT phenotype, decreased invasiveness, up-regulation of E-cadherin and enhanced spheroid formation. These results are in agreement with E-cadherin dependent sphere formation that we observe in breast cancer cell lines.

This is a clear dissociation between spheroid formation and the absolute reflection of the stem cell phenotype, suggesting that the spheroid assay may only be used as a positive assay to measure stem cell behavior in cell lines that express appropriate adhesion determinants in its surface, such as E-cadherin in breast epithelial carcinoma cells. In conclusion, our results support the use of the spheroid assay as suitable for the study of stem cell traits in breast cancer cell lines showing an epithelial phenotype and expressing proper cell surface molecules (such as E-cadherin), though this assay is non-informative on cell lines that lack the expression of E-cadherin, either due to physiological reasons (such as cells on a clear mesenchymal phenotype as MDA-MB-231 cells) or mutational driven (such as deletion of E-cadherin gene region in SKBR3 cells).

## Supporting Information

Figure S1 **Expression of surface markers in LTSF (A) and STSF and NSF (B) breast cancer cell lines.** Expression of 7 surface markers used to isolate mammary stem cells or breast cancer stem cells in the literature was checked by FACS. Isotypic control is represented by the grey curve and the specific staining by the white curve.
(TIF)

Figure S2 **Sphere formation in clonogenic conditions.** A) Quantitative limiting dilution assay for estimation of sphere forming cells in the population. Limiting dilution assay was performed as described in [9], essentially, different cell numbers were plated in 96 well plates (as depicted in left panel) under sphere forming conditions (see Mat. & Met.) and wells scored for the presence or not of spheres. Given the number of wells that effectively render sphere cultures a mathematical ELDA algorithm was used to estimate sphere forming cell frequency (plotted in right panel). B) Sphere formation from single cells. MCF7 cells were diluted to 1 cell per well in 96 well plates and sphere formation tracked over time. Seven typical clones are shown.
(TIF)

Figure S3 **CD44+/CD24-/low phenotype in breast cancer cell lines.** The expression of CD44 and CD24 markers was tested by FACS for each cell line and plotted and the percentage of each population is shown.
(TIF)

Figure S4 **ALDH1 activity in breast cancer cell lines.** ALDH1 activity was measured using the AldeFluor assay by FACS for each cell line and plotted, the percentage of ALDH1 positive cells is shown.
(TIF)

File S1 **List of antibodies used.**
(DOC)

File S2 **Gene list of the differentially expressed transcripts between LTSF, STSF and NSF cell lines.**
(XLSX)
