# Supplementary figures and images for: Mammosphere Formation in Breast Carcinoma Cell Lines Depends upon Expression of E-cadherin
Source: PLoS One. 2013 Oct 4;8(10):e77281. doi: 10.1371/journal.pone.0077281 (PMC3790762; doi:10.1371/journal.pone.0077281)

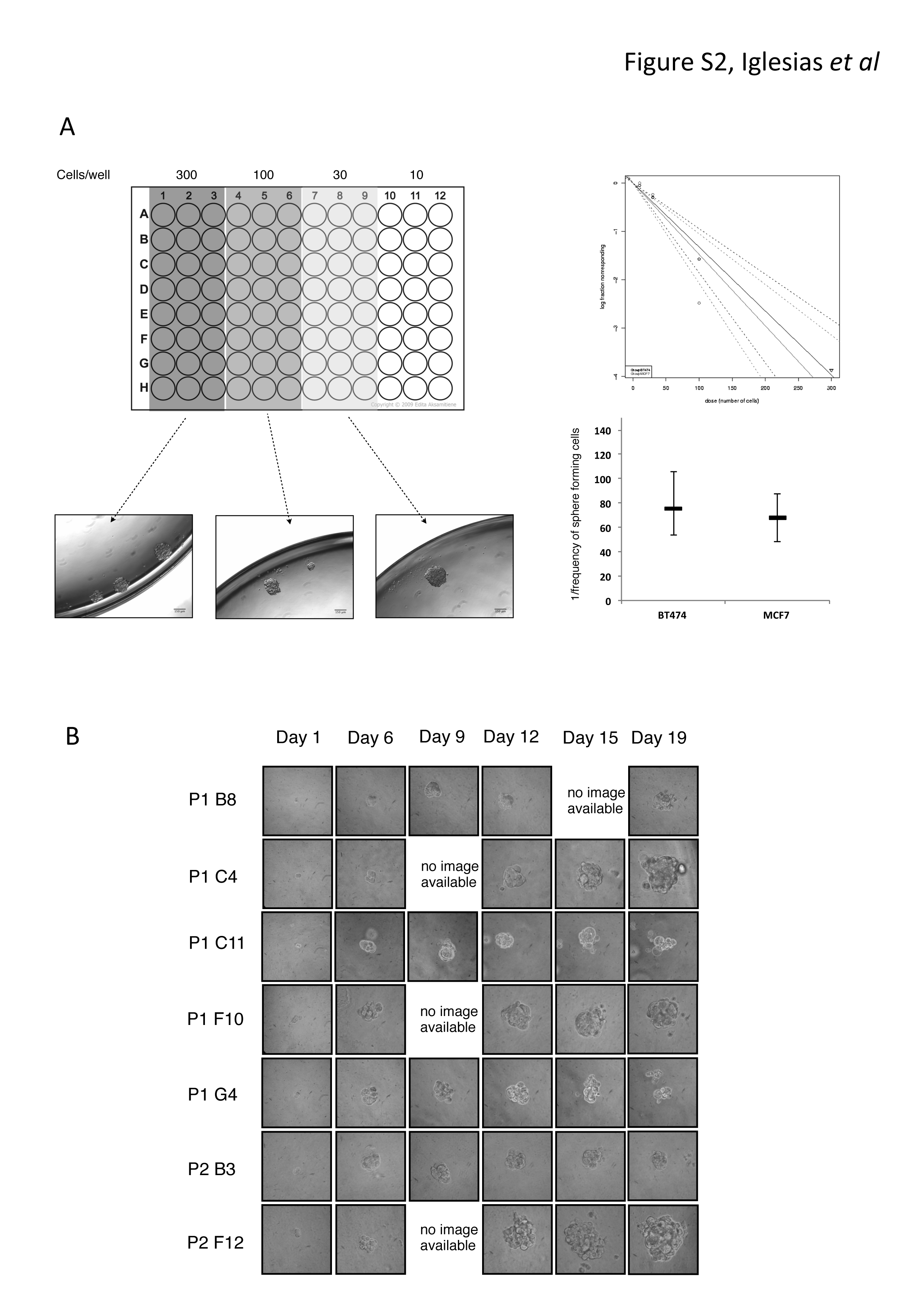

Supplement: Figure S2 — Sphere formation in clonogenic conditions. A) Quantitative limiting dilution assay for estimation of sphere forming cells in the population. Limiting dilution assay was performed as described in [9], essentially, different cell numbers were plated in 96 well plates (as depicted in left panel) under sphere forming conditions (see Mat. & Met.) and wells scored for the presence or not of spheres. Given the number of wells that effectively render sphere cultures a mathematical ELDA algorithm was used to estimate sphere forming cell frequency (plotted in right panel). B) Sphere formation from single cells. MCF7 cells were diluted to 1 cell per well in 96 well plates and sphere formation tracked over time. Seven typical clones are shown. (TIF) [file pone.0077281.s002.tif]

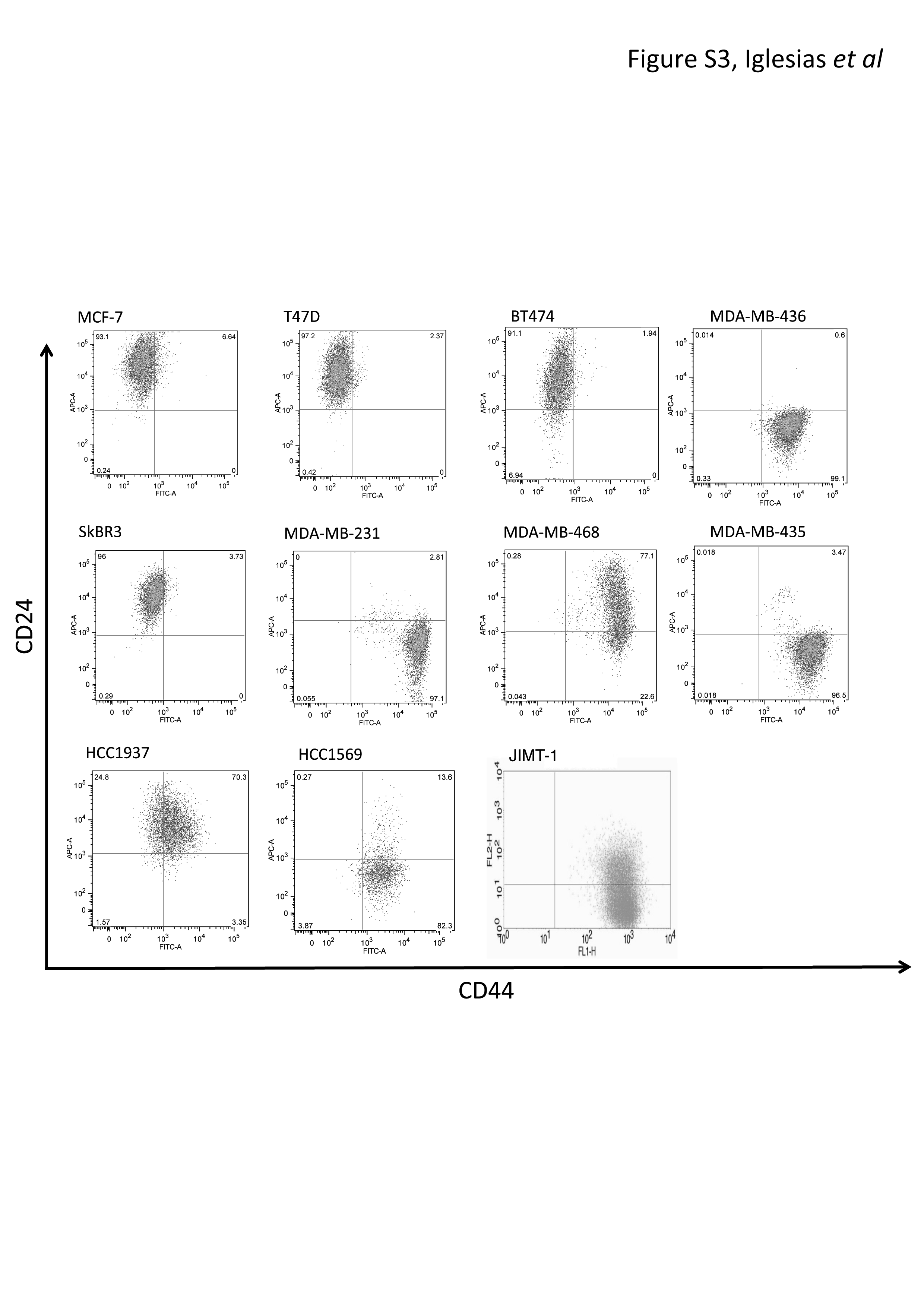

Supplement: Figure S3 — CD44+/CD24-/low phenotype in breast cancer cell lines. The expression of CD44 and CD24 markers was tested by FACS for each cell line and plotted and the percentage of each population is shown. (TIF) [file pone.0077281.s003.tif]

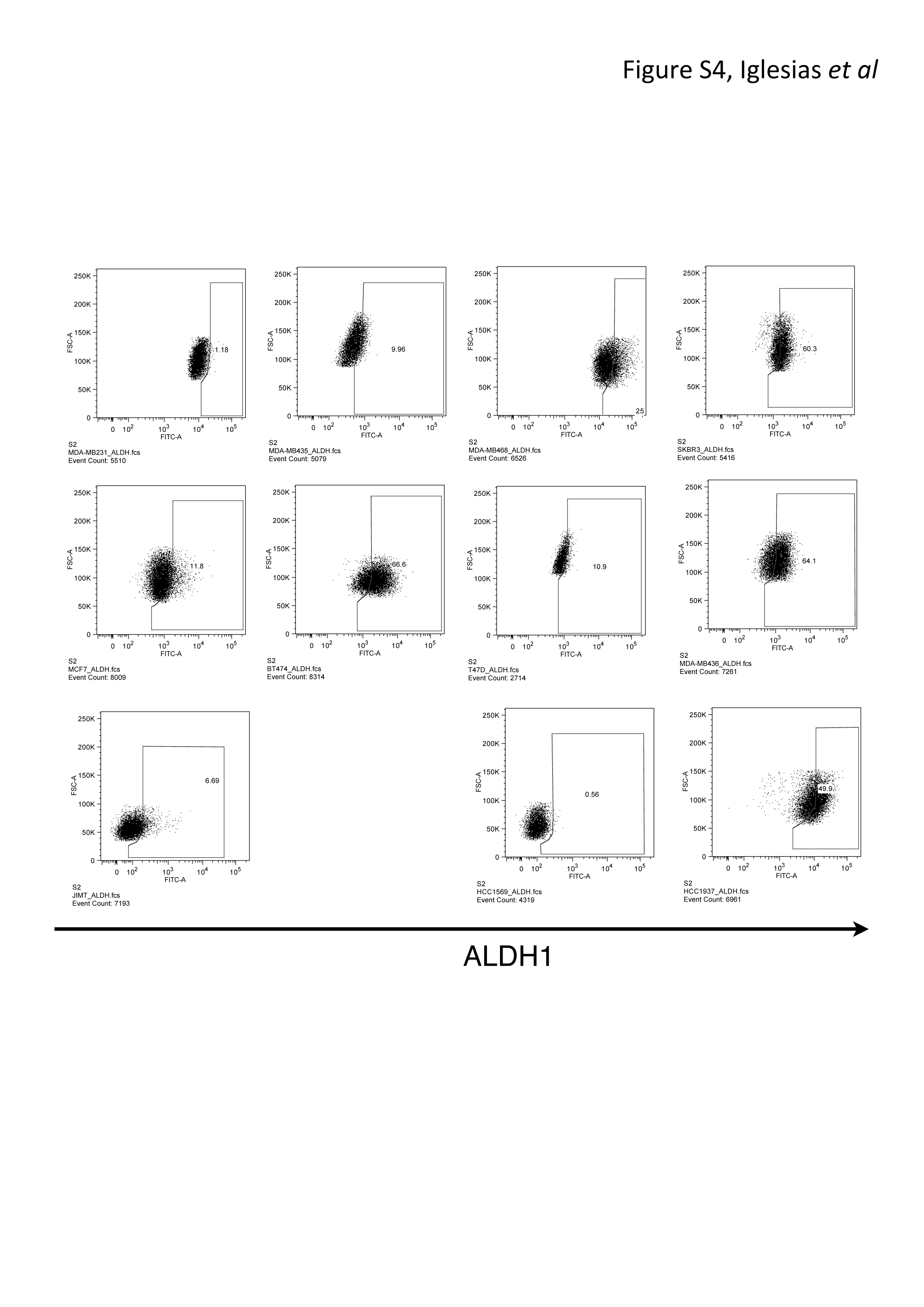

Supplement: Figure S4 — ALDH1 activity in breast cancer cell lines. ALDH1 activity was measured using the AldeFluor assay by FACS for each cell line and plotted, the percentage of ALDH1 positive cells is shown. (TIF) [file pone.0077281.s004.tif]
