# Supplementary material for: Mammosphere Formation in Breast Carcinoma Cell Lines Depends upon Expression of E-cadherin
Source: PLoS One. 2013 Oct 4;8(10):e77281. doi: 10.1371/journal.pone.0077281 (PMC3790762; doi:10.1371/journal.pone.0077281)
Supplement: File S1 — List of antibodies used. (DOC) [file pone.0077281.s005.doc]

Supp. File 1 Iglesias *et al*.

Supplementary File 1. List of antibodies used

**Antigen Clone Fluorochrome Manufacturer**

CD24 ML5 PE BD Biosciences

CD44 G44-26 FITC BD Biosciences

CD10 HI10a PE BD Biosciences

CD49f GoH3 FITC/PE BD Biosciences

EpCAM 9C4 APC Biolegend

CD29 MAR4 APC BD Biosciences

Muc1 HMPV FITC BD Biosciences

E-cadherin 24E10 Cell Signaling Technology

Anti-rabbit Alexa-488 Molecular Probes
